# Recruitment of γδ T cells to the lesion via the CCL2/CCR2 signaling after spinal cord injury

**DOI:** 10.1186/s12974-021-02115-0

**Published:** 2021-03-02

**Authors:** Ping Xu, Feng Zhang, Min-min Chang, Cheng Zhong, Cheng-Hong Sun, Hao-Ran Zhu, Jing-Chun Yao, Zhi-Zhong Li, Si-Tao Li, Wen-Cai Zhang, Guo-Dong Sun

**Affiliations:** 1 Department of Orthopedics, The First Affiliated Hospital of Jinan University, 601 West Whampoa Avenue, Guangzhou, 510000 China; 2 Intensive Care Unit, First Affiliated Hospital, Jinan University, Guangzhou, China; 3 College of Traditional Chinese Medicine, Jinan University, Guangzhou, China; 4 Department of Orthopedics, The Affiliated Jiangmen Traditional Chinese Medicine Hospital of Jinan University, Jiangmen, China; 5 State Key Laboratory of Generic Manufacture Technology of Chinese Traditional Medicine, Lunan Pharmaceutical Group Co. Ltd., Linyi, China; 6 Heyuan Affiliated Hospital of Jinan University, 733 Wenxiang Road, Heyuan, 517000 China; 7 Department of Pediatrics, The Sixth Affiliated Hospital, Sun Yat-sen University, No. 26 Yuancun Erheng Road, Tianhe District, Guangzhou, 510655 China

**Keywords:** Spinal cord injury, Inflammation, γδ T cell, CCL2, CCR2

## Abstract

**Background:**

Immune cell infiltration and neuroinflammation are heavily associated with spinal cord injury (SCI). C-C motif chemokine ligand 2/C-C chemokine receptor type 2 (CCL2/CCR2) axis has been identified as a critical role player during the invasion of immune cells to lesions in many diseases. γδ T cells, a subgroup of T cells, manage the course of inflammation response in various diseases; however, it remains unknown whether γδ T cells are recruited to injury site through CCL2/CCR2 signaling and exert the regulation effect on neuroinflammation after SCI.

**Methods:**

Basso Mouse Scale (BMS), regularity index, cadence, max contact area, and motor-evoked potential testing (MEP) were measured to determine the neurological function recovery after spinal cord injury. Nissl staining was performed to identify the number of surviving motor neurons at lesion epicenter. Immunofluorescence, Western blot, enzyme-linked immunosorbent assays (ELISA), and quantitative real-time polymerase chain reaction (QRT-PCR) also were employed to evaluate the expression of associated proteins and genes.

**Results:**

In this study, we demonstrated that TCRδ^−/−^ mice present improved neurological recovery after SCI. γδ T cell recruitment to the SCI site was significantly reduced and motor functional improvement enhanced in CCL2^−/−^ and CCR2^−/−^ mouse strains. Furthermore, reconstitution of TCRδ^−/−^ mice with γδ T cells extracted from CCR2^−/−^ mice also showed similar results to CCL2 and CCR2 deficient mice.

**Conclusions:**

In conclusion, γδ T cell recruitment to SCI site promotes inflammatory response and exacerbates neurological impairment. CCL2/CCR2 signaling is a vital recruitment mechanism of γδ T cells to the SCI site, and it may be taken as a novel therapeutic target for future SCI.

**Supplementary Information:**

The online version contains supplementary material available at 10.1186/s12974-021-02115-0.

## Introduction

Spinal cord injury (SCI) can cause neurological dysfunctions such as irreversible loss of sensory and motor functions below individual injury levels [1]. Moreover, it is characterized by high rates of morbidity and disability, which to a large extent, increase the difficultly of treatment for SCI [2]. Although the treatment of SCI involves general measures such as resuscitation, and specific treatment that also varies between early and late and that related to the insult itself or consequence/complications, the clinical efficiency of those measures is very limited [3, 4]. We, therefore, need to urgently perform further research on the pathological mechanism of SCI for developing more effective treatments.

The pathological progression of SCI is divided into two parts: primary injury and secondary injury. The primary injury refers to mechanical injuries to local tissues and vessels including compression, contusion, and stretch and tear [5]. In addition, the primary injury can induce inflammatory immune responses that are closely associated with the secondary injury. Inflammation increases the permeability of the blood-spinal cord barrier, further facilitating the infiltration of peripheral immune cells such as γδ T cells and macrophages to the injury site, thus adding to the existing aggravated damage. γδ T cells represent a subset of T cells that mainly exist in mucous membranes, skin, and adipose tissue. Moreover, they are MHC-unrestricted innate-like lymphocytes with unique antigen receptors, and play a vital role in innate and immune responses [6, 7]. In addition, γδ T cells not only respond quickly to infection or inflammation but also promote secretion of IFN-γ, IL-17, and IL-23 that might seriously influence the outcome of central nervous system diseases [8]. Our previous study showed that γδ T cells were recruited to the lesion as a main source of IFN-γ in the early stage after SCI, which induced macrophage polarization into M1 phenotype, released pro-inflammatory cytokines, and further hampered recovery of the injury cord [4]. However, the mechanism by which γδ T cells migrate to the site of SCI is still unknown.

**Scheme 1:**
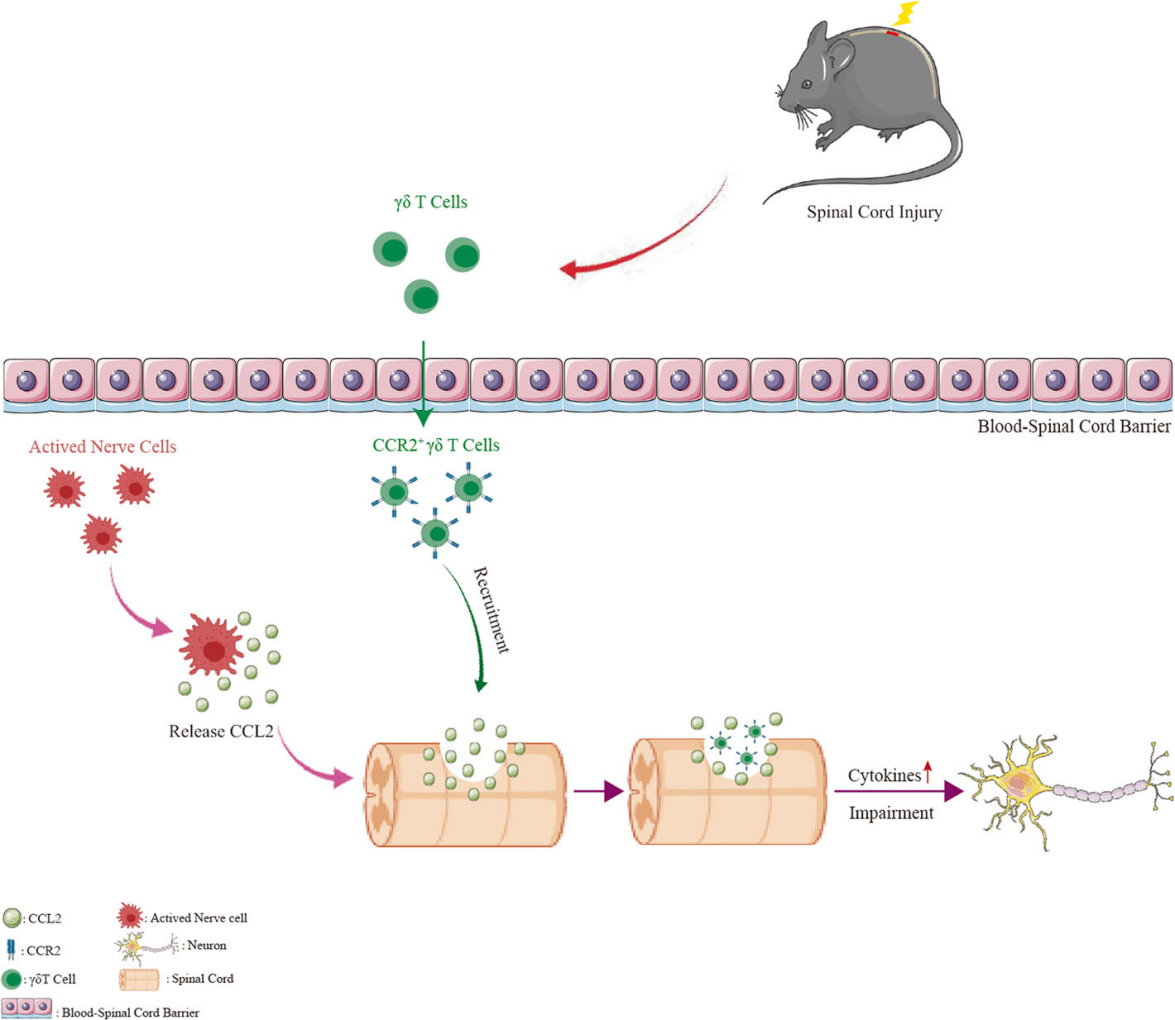
Schematic illustration for the recruitment mechanism of γδ T cells to the site of spinal cord injury

C-C motif chemokine ligand 2 (CCL2/MPC-1) is a chemokine that recruits immune cells to inflammatory site through blinding to C-C chemokine receptor type 2 (CCR2) [9]. A recent study demonstrated that CCX872, a selective CCR2 antagonist, could decrease the accumulation of M1-type macrophages, and further improve the cognitive dysfunction caused by traumatic brain injury [10]. Both CCR2 and CCR6 are expressed on the surface of γδ T cells and can efficiently regulate the directional migration of γδ T cells. However, under inflammatory conditions, the expression of CCR6 is downregulated, leading to the aggregation of γδ T cells to the inflammation site mainly through CCR2 [8, 11]. Previous studies indicated that CCR2 induced the accumulation of γδ T cells in inflammatory skin caused by psoriatic arthritis and those cells mainly exhibited the CCR6^-^-CCR2^+^ phenotype [12, 13]. Other studies have demonstrated that CCR2, rather than CCR6, is a key driver of γδ T cell recruitment to the central nervous system in encephalomyelitis models [14]. Collectively, we proposed the following hypothesis: γδ T cells may be recruited to the injury site via CCL2/CCR2 signaling after SCI (Scheme 1).

In order to test this hypothesis, we performed a comprehensive research to investigate the recruitment mechanism of γδ T cells to the lesion after SCI, by utilizing TCRδ^−/−^, CCL2^−/−^, and CCR2^−/−^ mice from Jackson Laboratory. Our results suggested that TCRδ^−/−^ mice showed improved function recovery after SCI. γδ T cell recruitment to the SCI site was significantly reduced and motor functional improvement was elevated in CCL2^−/−^ and CCR2^−/−^ mice. Additionally, reconstitution of TCRδ^−/−^ mice using γδ T cells extracted from CCR2^−/−^ mice also showed similar results to CCL2 and CCR2 deficient mice.

## Methods and materials

### Animals

C57bL/6 (No. 000664), TCRδ^−/−^ (No. 002120), CCL2^−/−^ (No. 004434), and CCR2^−/−^ (No. 004999) mice were purchased from the Jackson Laboratory. All mice were females with average age of 6–8 weeks old and weight of 17–22 g at time of surgery; they were maintained under pathogen-free conditions in the animal facility at Jinan University. All procedures were approved by and performed in accordance with Jinan University’s Institutional Laboratory Animal Care and Use Committee.

### Cerebrospinal fluid collection from patients

Patients suffering acute SCI were recruited at The First Affiliated Hospital of Jinan University by spinal surgeons from June 2016 to July 2020. Patients met by the following criteria were included: (1) American Spinal Injury Association (ASIA) grade A or B SCI upon presentation; (2) the lesion segment was between C4 and T10 inclusive; (3) within 48 h after SCI and the ability to complete appropriate neurological examination. The excluded criteria included (1) concomitant head injuries; (2) concomitant serious chest trauma, pelvis, or extremities that required surgical intervention (e.g., chest tube, internal or external fixation); and (3) without ability to undergo a reliable neurological examination. Myelotomy was performed by experienced surgeons to achieve the cerebrospinal fluid (CSF) from SCI patients. In order to obtain the control samples from non-SCI patients, we enrolled individuals who had knee osteoarthritis and underwent knee replacements under spinal anesthesia. A 1.5–2.0-ml sample of CSF was collected with a spinal needle that used to punctured the dura. After that, the anesthetic agent was injected. The clinical experiment was approved by the Human Ethics Committee of Jinan University, and the CSF collection was approved by patients.

### Regents

Anti-mouse CCL2 (#2029S) was purchased from Cell Signaling Technology. Anti-mouse CCR2 (ab203128) and TCR-γ/δ (ab231545) were purchased from Abcam Co., LTD. The enzyme-linked immunosorbent assays (ELISA) kits were purchased from Invitrogen Co., LTD. RNAeasy™ Animal RNA Isolation Kit was purchased from Beyotime Co., LTD. ReverTra Ace qPCR RT Kit and SYBR® Green Realtime PCR assay kits were purchased from TOYOBO Co., LTD.

### Contusion model of spinal cord

SCI model was completed by use of New York University impactor as previously described [15]. Firstly, mice were anesthetized by the peritoneal injection of 7% chloral hydrate, and then underwent a laminectomy at the T11 and T12. After stabilizing the spine, a l0-g rod was released to hit the exposed dorsal surface of the cord from a height of 6.25 mm. Subsequently, muscles and skin were stitched in layers, and after mice underwent surgery were maintained in a suitable environment. Manual bladder emptying was completed twice daily until the recovery of reflex bladder emptying.

### Tissue processing

After deep anesthetization using Chloral hydrate (7 mg/kg), 20 ml PBS was transcardially perfused, followed by 4% paraformaldehyde (PFA). A 5 mm segment of cord centered on T11 was then removed and quickly soaked in 4% PFA at 4° overnight; subsequently, that segment was transferred into 15% sucrose solution until the tissues sank. This was followed by 30% sucrose solution using the same procedure.

### Histology and immunofluorescence

The 15-μm-thick serial frozen sections of spinal cords were stained with 0.1% cresyl violet to image surviving neurons at lesion epicenter. For immunofluorescence, frozen samples were cut into 10 μm sections (Leica). Slices were permeabilized by 0.2% Triton X-100 (Sigma-Aldrich), then incubated with the following mouse-specific primary antibodies overnight: 1:100 anti-CCR2 and 1:50 anti-TCR-γ/δ. Subsequently, both or single staining were performed using 1:1000 fluorescent Alexa Fluor 488 or 546 secondary antibodies (Invitrogen Vector) at room temperature for 1.5 h. After PBST (0.2% Tween-20 in PBS) washing, the tissue slices were fixed with Vectashield containing DAPI and used for visualization. In order to capture images at lesion site and perform further quantification analysis, we used an inverted fluorescence microscope (Axio Observer A1; Carl Zeiss) and ImageJ software.

### Western blot

Radioimmunoprecipitation lysis buffer with 2 mg/ml aprotinin and 1 mM PMSF was used to extract lysates from fresh tissues or cells. Then these lysates were subjected to 10,000 g centrifugation at 4 °C for 20 min. The concentration of extracted protein was assessed using bicinchoninic acid protein assay kit. Finally, immunoblotting was conducted according to reported previously [16].

### ELISA

For mouse, CCL2 (Invitrogen Catalog 88-7391-77) and inflammatory cytokines concentrations extracted from fresh injury cord were quantified by associated ELISA kit. For human, the content of MCP-1 in cerebrospinal fluid (CSF) of SCI or non-SCI patients was measured by the same method as above (Invitrogen Catalog 88-7399-22). Fresh samples from each experiment were tested in triplicate in accordance with the manufacturer's instructions.

### Quantitative real-time polymerase chain reaction

We used RNAeasy™ Animal RNA Isolation Kit (Beyotime) to extract RNA from fresh spinal cord tissue. The extracted RNA was denatured at 65 °C for 5 min and immediately cooled, followed by synthetic of cDNA with ReverTra Ace qPCR RT Kit (TOYOBO). qRT-PCR primers for the genes listed in Additional file 1: Table S1 were purchased from Shanghai Generay Biotech Co., LTD., after which, SYBR® Green Realtime PCR assay kit (TOYOBO) was used to perform qRT-PCR. Each sample was performed in triplicate. Data were normalized to the reference gene (GAPDH) and expressed as fold change.

### Basso Mouse Scale

The Basso Mouse Scale (BMS) for locomotion is based on a scale ranging from 0 (no ankle movement) to 9 (complete normality) [17]. The mice were kept in an open area surrounded by plates for 4 min. Hind limb motor ability was independently assessed by two observers and the mean locomotion score was considered the individual’s BMS by both.

### CatWalk-assisted gait analysis

At 6 weeks post-injury, an automatic quantitative gait analysis system (CatWalk XT; Noldus) was used to evaluate the gait of mice from each group. Each mouse required continuous walking along a 50 cm path on a glass plate, and was completed at least three times. The CatWalk system automatically recognized and marked each paw print, then generated a series of parameters involving paw statistics, mean speed and cadence, step sequence, and basics of support. The walking coordination was evaluated using the regularity index as described previously [18], calculated as the number of normal step sequence patterns multiplied by four and divided by the total amount of paw placements; that value is ~ 100% in normal animals. The max contact area refers to the maximal area of contact between the paw and the walking floor.

### Motor-evoked potential testing

The motor-evoked potential testing (MEPs) of mice were assayed by electromyography at the 6 weeks post-injury as described before [19], with minor modifications. A stimulating electrode was placed in the rostral end of surgical exposure area and a recording electrode was placed in their bicep flexor muscles. Subsequently, a single square wave stimulus of 0.5 mA was used, with a 0.5 ms duration, a delay of 2 ms, and 1 Hz. Measuring the amplitude from the initiation point of the first reaction wave to its highest point, the nerve conduction function in the hind limb was indicated by the peak-to-peak amplitude (*P*-*P* value).

### γδ T cell proliferation and transplantation

In order to determine the proliferation of splenic γδ T cells from WT or CCR2^−/−^ mice (*n* = 10 mice/per group) in vitro, splenic γδ T cells were activated by purified anti-mouse TCR-δ (10 μg/ml) and soluble anti-CD28 antibody (1 μg/ml) and expanded for 6 days in the presence of IL-2 (2 ng/ml) as previously described [20–22]. Then, expanded cells were magnetic-activated cell sorting (MACS) sorted by biotin-conjugated anti-TCR-δ antibody and used for subsequent experiments (99% purity). For transplantation of cells, the mice were immobilized by a tail vein injection fixator. Then, splenic γδ T cells from WT or CCR2^−/−^ mice were injected intravenously into TCRδ^−/−^ mice (2 × 10^6^ cells/per mouse). One day after the transplantation, all mice were subjected to SCI, and ethology evaluation followed.

### Statistical analysis

The Prism 7 software (Graphpad) was used to process and analyze all data. Values were presented as mean ± standard difference (SD) or standard error of mean (SEM). A two-way ANOVA was performed to test the significance in BMS score between groups over time. Remaining data were analyzed using Student’s *t* test or one-way ANOVA with Tukey’s multiple comparison test. Values were regarded as significant at *P* < 0.05.

## Results

### γδ T cells play a detrimental role in SCI

**Fig. 1:**
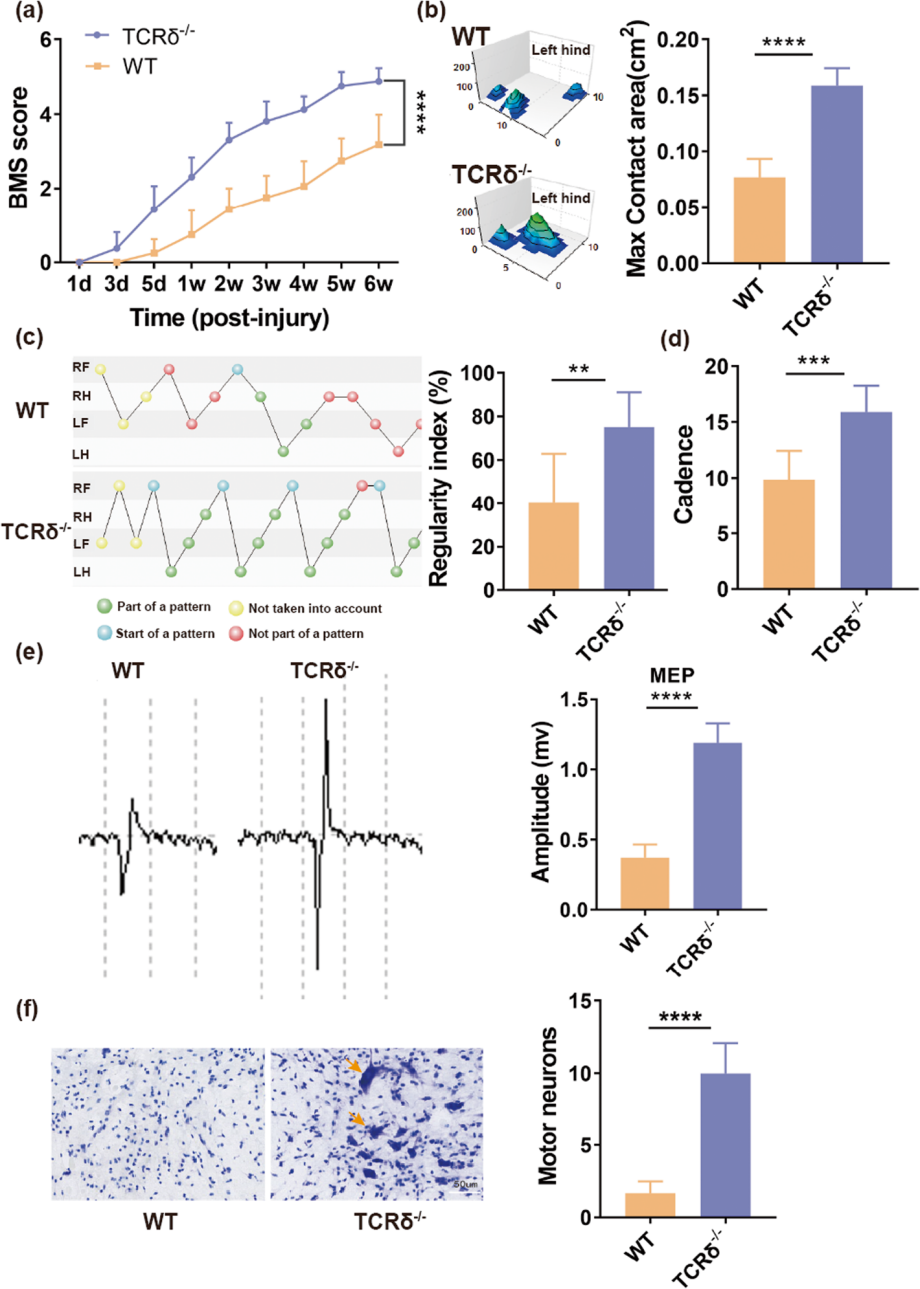
γδ T cells play a detrimental role in spinal cord injury (SCI). **a** BMS score of WT and TCRδ^−/−^ mice at different time points after spinal cord contusion (*n* = 8 mice/per group). **b** Max contact area of WT and TCRδ^−/−^ mice at 6 weeks after SCI (*n* = 8 mice/per group). **c** Regularity index of WT and TCRδ^−/−^ mice at 6 weeks after SCI (*n* = 8 mice/per group). **d** Cadence of WT and TCRδ^−/−^ mice at 6 weeks after SCI (*n* = 8 mice/per group). **e** Motor-evoked potential (MEP) recordings from WT and TCRδ^−/−^ mice at 6 weeks post-surgery (*n* = 8 mice/per group). **f** Survival of motor neurons immunostained with Nissl staining in cross-section of spinal cord lesion epicenter at 6 weeks after SCI (*n* = 6 mice/per group). **P < 0.01; ***P < 0.001; ****P < 0.0001

We performed a moderate contusion at the T11 levels of TCRδ^−/−^ and age-matched WT mice; the spontaneous improvement of hind limb motor ability was evaluated through the BMS. Our results suggested that both TCRδ^−/−^ and WT mice groups presented complete hind limb paralysis with a BMS score of 0 at 1 day post-injury. Beginning from 3 days post-injury, TCRδ^−/−^ mice recovered gradually, and their BMS scores increased progressively (Fig. 1a). By comparison, motor improvement in WT mice group was obviously slower, and their mean BMS score was significantly lower than those of TCRδ^−/−^ mice group at 6 weeks post-injury (3.19 ± 0.8 vs. 4.89 ± 0.35, *P* < 0.0001, Fig. 1a, *n* = 8 mice/per group). In terms of max contact area, regularity index, and cadence, compared with WT mice group, TCRδ^−/−^ mice group had a larger mean max contact area (0.16 ± 0.02 vs. 0.08 ± 0.02 cm^2^, *P* < 0.0001, Fig. 1b, *n* = 8 mice/per group), a higher mean regularity index (75.23 ± 15.99% vs. 40.29 ± 22.61%, *P* < 0.01, Fig. 1c, *n* = 8 mice/per group), and greater mean cadence (15.9 ± 2.36 vs. 9.89 ± 2.55, *P* < 0.001, Fig. 1d, *n* = 8 mice/per group) at 6 weeks after injury. To further confirm this, electromyography of biceps flexor cruris was recorded through stimulating the dura mater at the T6 level at 6 weeks post-injury. Our results demonstrated that a higher mean amplitude of motor-evoked potentials (MEPs) was found in TCRδ^−/−^ mice group than in WT mice group (1.19 ± 0.14 vs. 0.37 ± 0.09 mV, *P* < 0.0001, Fig. 1e, *n* = 8 mice/per group), indicating a better improvement of motor function of injury to hind limbs in TCRδ^−/−^ mice group compared to WT mice group. In addition, to evaluate whether motor neurons of TCRδ^−/−^ mice group were better protected than those of WT mice group, we performed a Nissl staining to measure the number of survival motor neurons of lesion epicenter in cross section at 6 weeks post-injury. Our results meant that more survival motor neurons were found in TCRδ^−/−^ mice compared with WT mice (10 ± 2.1 vs. 1.67 ± 0.82, *P* < 0.0001, Fig. 1f, *n* = 6 mice/per group). All above results suggested that assemblage of γδ T cells resulted in the aggravation of lesions and hampered function recovery after SCI.

### Increased secretion of CCL2 after SCI

**Fig. 2:**
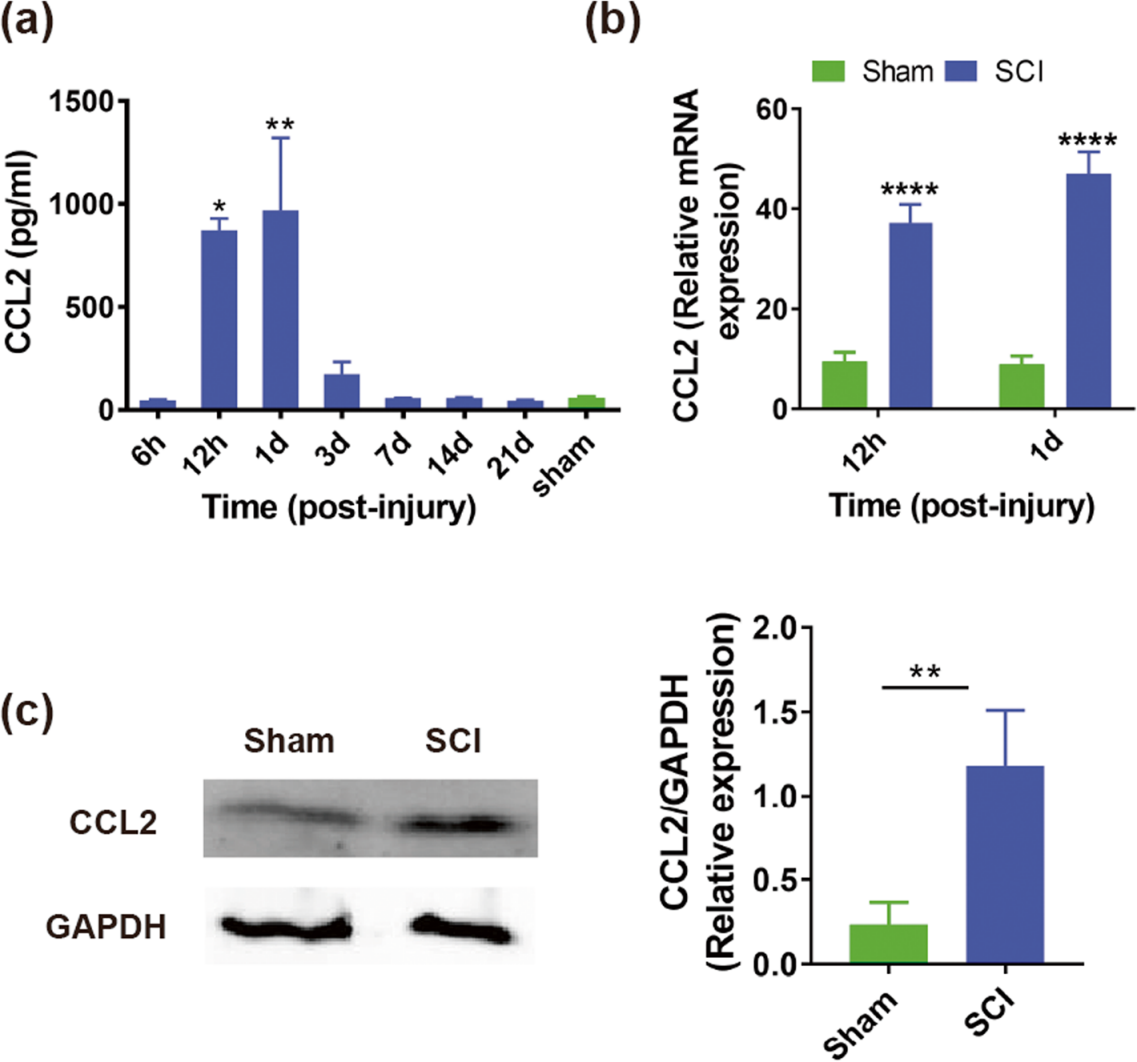
Increased secretion of CCL2 after spinal cord injury (SCI). **a** The levels of CCL2 was significantly increased at 12 h and 1 day after SCI compared with that of sham group (*n* = 3 mice/per group). **b** QRT-PCR quantitative analysis of CCL2 expression at 12 h and 1 day after SCI and sham (*n* = 8 mice/per group). **c** The protein level of CCL2 was obviously higher in SCI group than that of sham group at 1 day after surgery (*n* = 3 mice/per group). *P < 0.05; **P < 0.01; ****P < 0.0001

**Fig. 3:**
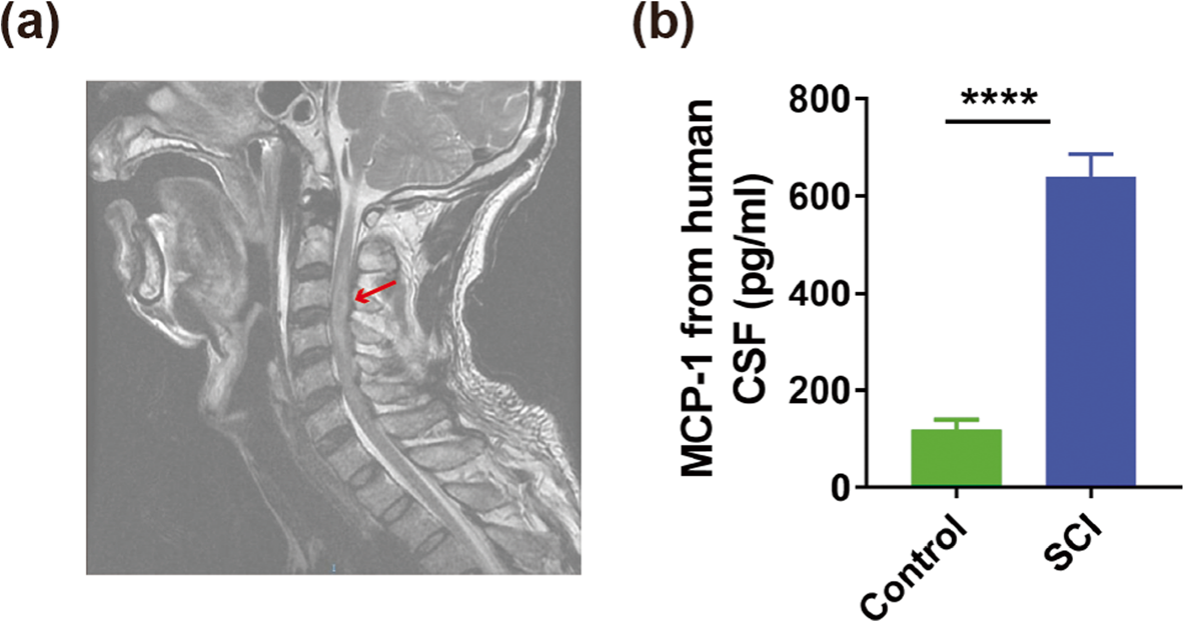
Increased secretion of MCP-1 after spinal cord injury (SCI) **a** magnetic resonance imaging of SCI patient. **b** The comparison of MCP-1 levels of cerebrospinal fluid (CSF) between SCI (*n* = 6) and control group (*n* = 20). *P < 0.05; **P < 0.01; ****P < 0.0001

To investigate whether amounts of CCL2 were secreted after SCI, we evaluated the level of CCL2 at different time points in the CSF after SCI or laminectomy (sham) using ELISA kit. Our results showed that the expression level of CCL2 increased significantly at 12 h to 1 day after SCI compared with sham group (12 h: 871.2 ± 99.58 vs. 58.33 ± 9.82 pg/ml, *P* < 0.05; 1 day: 967.5 ± 611.6 vs. 58.33 ± 9.82 pg/ml, *P* < 0.01, Fig. 2a, *n* = 3 mice/per group). Additionally, the gene expression of CCL2 in the associated cord at 12 h and 1 day after injury or laminectomy was evaluated through quantitative real-time polymerase chain reaction (QRT-PCR) testing. Our results demonstrated that a higher gene expression of CCL2 was found in SCI group than in sham group (12 h: 37.2 ± 3.65 vs. 9.45 ± 1.85 pg/ml, *P* < 0.0001; 1 day: 47.03 ± 4.38 vs. 8.95 ± 1.67 pg/ml, *P* < 0.0001, Fig. 2b, *n* = 8 mice/per group). Meanwhile, we also performed an immunoblotting to evaluate the level of CCL2 protein in spinal cord after SCI or laminectomy. Our results demonstrated that SCI group had a greater CCL2 protein level compared with sham group (1.18 ± 0.33 vs. 0.23 ± 0.13, *P* < 0.01, Fig. 2c, *n* = 6 mice/per group). To further identify this, we also assessed the level of MCP-1 in the CSF of SCI patients (Fig. 3a, *n* = 6) at 1 day after injury or control group (*n* = 20). We found that compared to control group, SCI patients group had significantly higher levels of MCP-1 in CSF (639.35 ± 46.48 vs. 119.05 ± 20.31 pg/ml, *P* < 0.0001, Fig. 3b). These outcomes indicated that amounts of CCL2/MCP-1 were released after SCI.

### High expression of CCR2 on the surface of γδ T cells

**Fig. 4:**
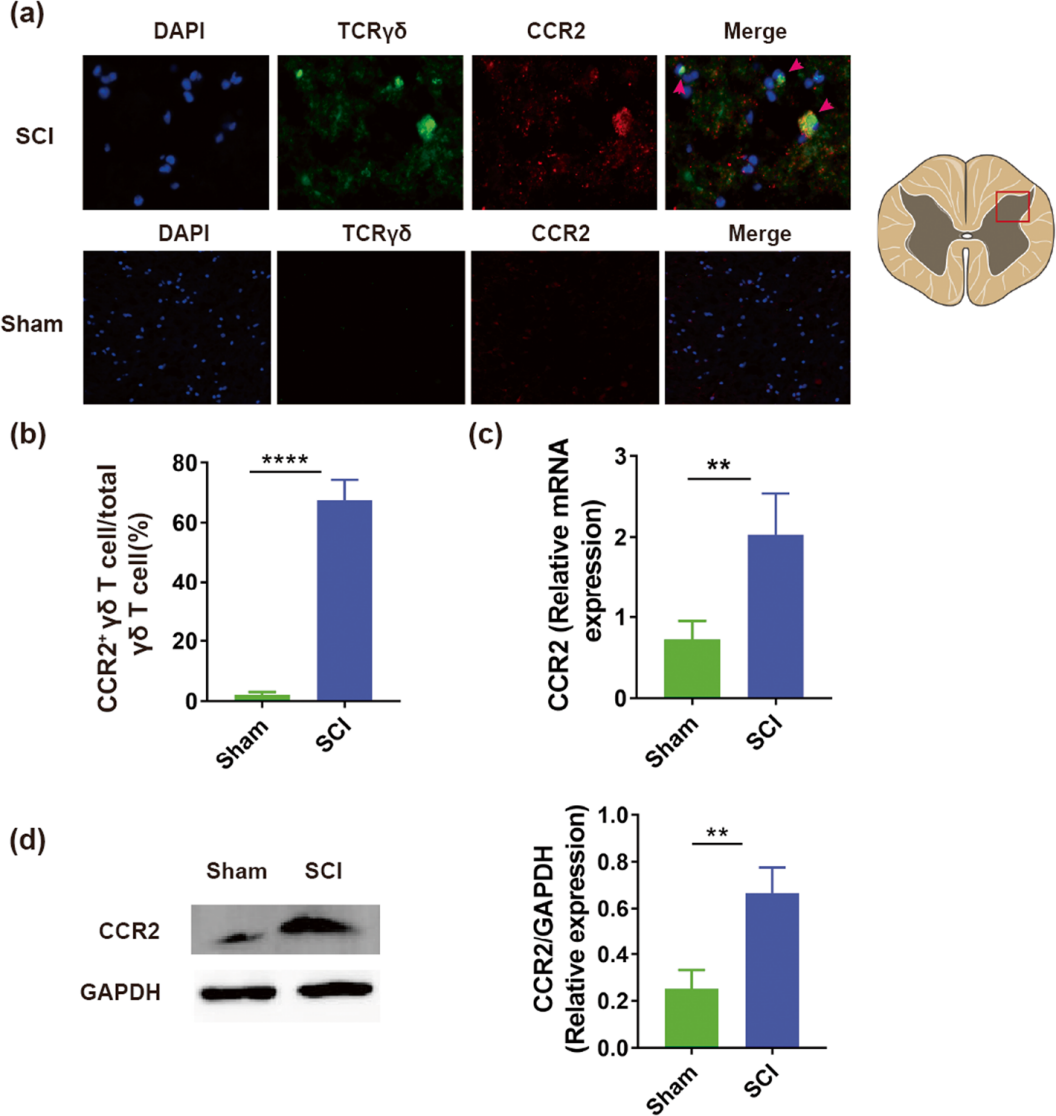
High expression of CCR2 on surface of γδ T cells after spinal cord injury (SCI). **a** Spinal sections from mice after 1d post-SCI and sham were immunostained with anti-TCRγδ (green, a maker for γδ T cell) and anti-CCR2 (red) (*n* = 6 mice/per group). **b** Statistic histogram of the percent of CCR2^+^ γδ T cell (CCR2^+^/total γδ T cell, *n* = 6 mice/per group). **c** The gene expression of CCR2 at 1d post-SCI and sham (*n* = 8 mic/per group). **d** Comparison of protein level of CCR2 between SCI and sham groups (1d post-surgery, *n* = 6 mice/per group). **P < 0.01; ****P < 0.0001

To confirm whether CCR2, a special receptor of CCL2, was highly activated on the surface of γδ T cells after SCI, we performed an immunofluorescence for γδ T cells and CCR2 in lesion epicenter cross-section of cord after SCI or laminectomy. We found that amounts of γδ T cells, highly presenting CCR2^+^, were recruited in SCI site, but laminectomy group did not exhibit this (67.5 ± 6.83% vs. 2.18 ± 0.82%, *P* < 0.0001, Fig. 4a, b, *n* = 6 mice/per group). Furthermore, the results of QRT-PCR showed that the gene expression of CCR2 in SCI group was much higher than that in sham group (2.03 ± 0.52 vs. 0.72 ± 0.23, *P* < 0.01, Fig. 4c, *n* = 8 mice/per group). To further confirm this, we conducted an immunoblotting to measure the expression level of CCR2 protein in spinal cord after SCI or laminectomy. Our results showed that a higher level of CCR2 was found in SCI group than in sham group (0.67 ± 0.11 vs. 0.25 ± 0.08, *P* < 0.01, Fig. 4d, *n* = 6 mice/per group). These outcomes suggested that CCR2 was highly activated on the surface of γδ T cells after SCI.

### CCL2 depletion contributes to decreased recruitment of γδ T cells at SCI site

**Fig. 5:**
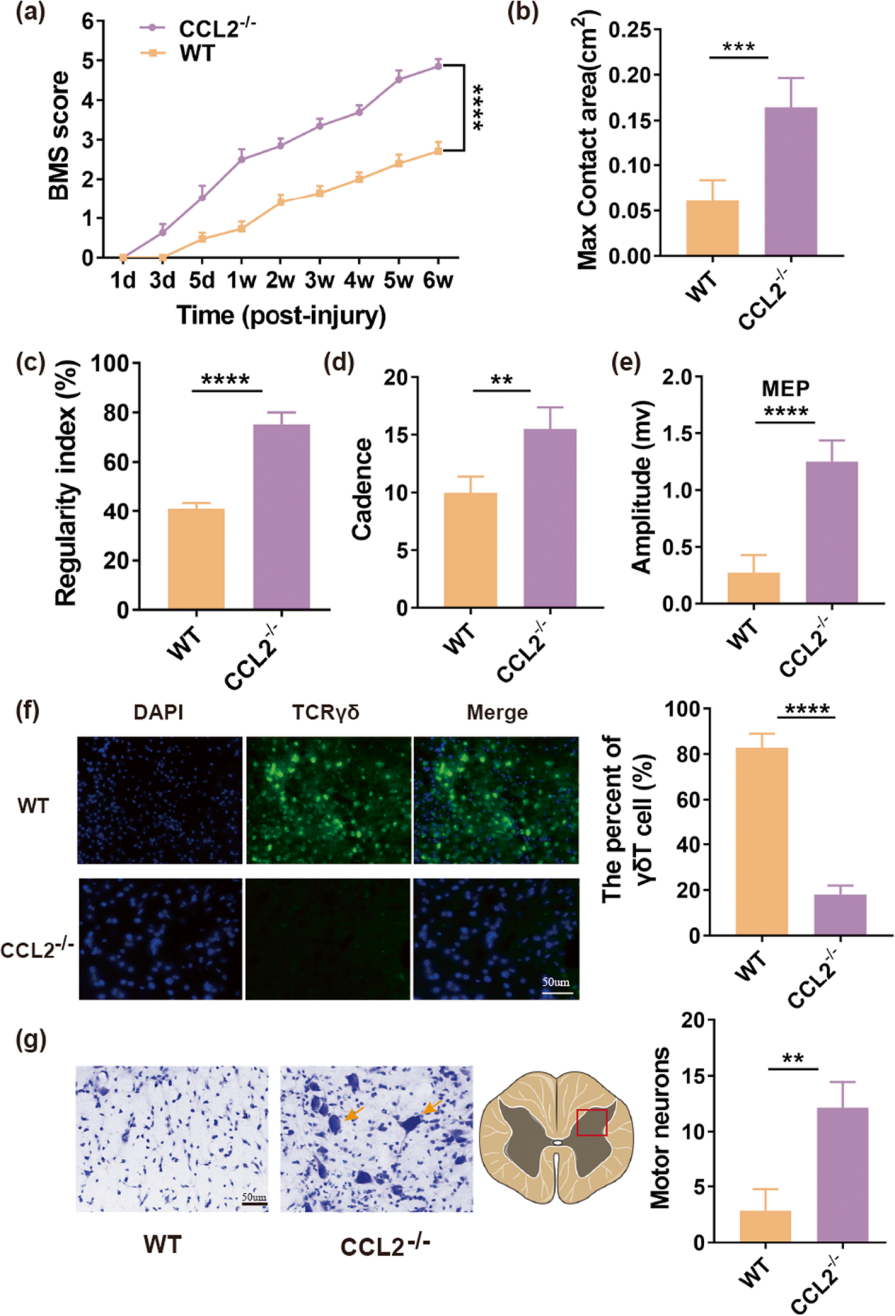
CCL2 plays an important role during recruitment of γδ T cells to lesions. **a** BMS score of WT and CCL2^−/−^ mice at different time points after spinal cord injury (SCI) (*n* = 8 mice/per group). **b** Max contact area of WT and CCL2^−/−^ mice at 6 weeks after SCI (*n* = 8 mice/per group). **c** Regularity index of WT and CCL2^−/−^ mice at 6 weeks after SCI (*n* = 8 mice/per group). **d** Cadence of WT and CCL2^−/−^ mice at 6 weeks after SCI (*n* = 8 mice/per group). **e** Motor-evoked potential (MEP) recordings from WT and CCL2^−/−^ mice at 6 weeks post-surgery (*n* = 8 mice/per group). **f** Spinal sections from WT and CCL2^−/−^ mice after 1 day post-SCI were immunostained with anti-TCRγδ (green, a maker for γδ T cell) and the corresponding static histogram of percent of γδ T cell (γδ T cells/total cells) (*n* = 6 mice/per group). **g** Survival of motor neurons immunostained with Nissl staining in cross-section of spinal cord lesion epicenter at 6 weeks after SCI (*n* = 6 mice/per group). **P < 0.01; ***P < 0.001; ****P < 0.0001

To define whether CCL2 plays a vital role during the migration of γδ T cells to lesions after SCI, we performed an immunostaining to evaluate the aggregation of γδ T cells at the site of lesions using anti-TCR-γ/δ in both CCL2^−/−^ and WT mice groups. Our results suggested that the CCL2^−/−^ mice group had significantly less γδ T cells at the epicenter site of injury compared to WT mice group (18 ± 4% vs. 82.83 ± 6.18%, *P* < 0.0001, Fig. 5f, *n* = 6 mice/per group). Meanwhile, we also assessed the BMS score, regularity index, cadence, and MEPs. We found that CCL2^−/−^ mice recovered progressively, and had a higher mean BMS score at 6 wk post-injury compared with WT mice (4.85 ± 0.18 vs. 2.72 ± 0.23, *P* < 0.0001, Fig. 5a, *n* = 8 mice/per group). This significant difference was also presented in an enlarged max contact area (0.17 ± 0.03 vs. 0.06 ± 0.02 cm^2^, *P* < 0.001, Fig. 5b) increased regularity index (75.17 ± 4.88% vs. 41 ± 2.61%, *P* < 0.0001, Fig. 5c), enhanced cadence (15.5 ± 1.87 vs. 10 ± 1.41, *P* < 0.01, Fig. 5d) and boosted MEPs (1.25 ± 0.19 vs. 0.28 ± 0.15 mv, *P* < 0.0001, Fig. 5e) in CCL2^−/−^ mice at 6 weeks post-injury, compared to WT mice (*n* = 8 mice/per group). In addition, a Nissl staining was performed to compare the number of survival motor neurons of lesion epicenter in cross-section between CCL2^−/−^ and WT mice groups at 6 weeks after injury. Our results demonstrated that more motor neurons were found in CCL2^−/−^ mice than in WT mice (12.17 ± 2.32 vs. 2.83 ± 1.94, *P* < 0.01, Fig. 5g, *n* = 6 mice/per group). Those outcomes indicated that depletion of CCL2 contributed to decreased aggregation of γδ T cells at the site of lesions and further promoted function recovery.

### CCR2 depletion contributes to decreased recruitment of γδ T cells at SCI site

**Fig. 6:**
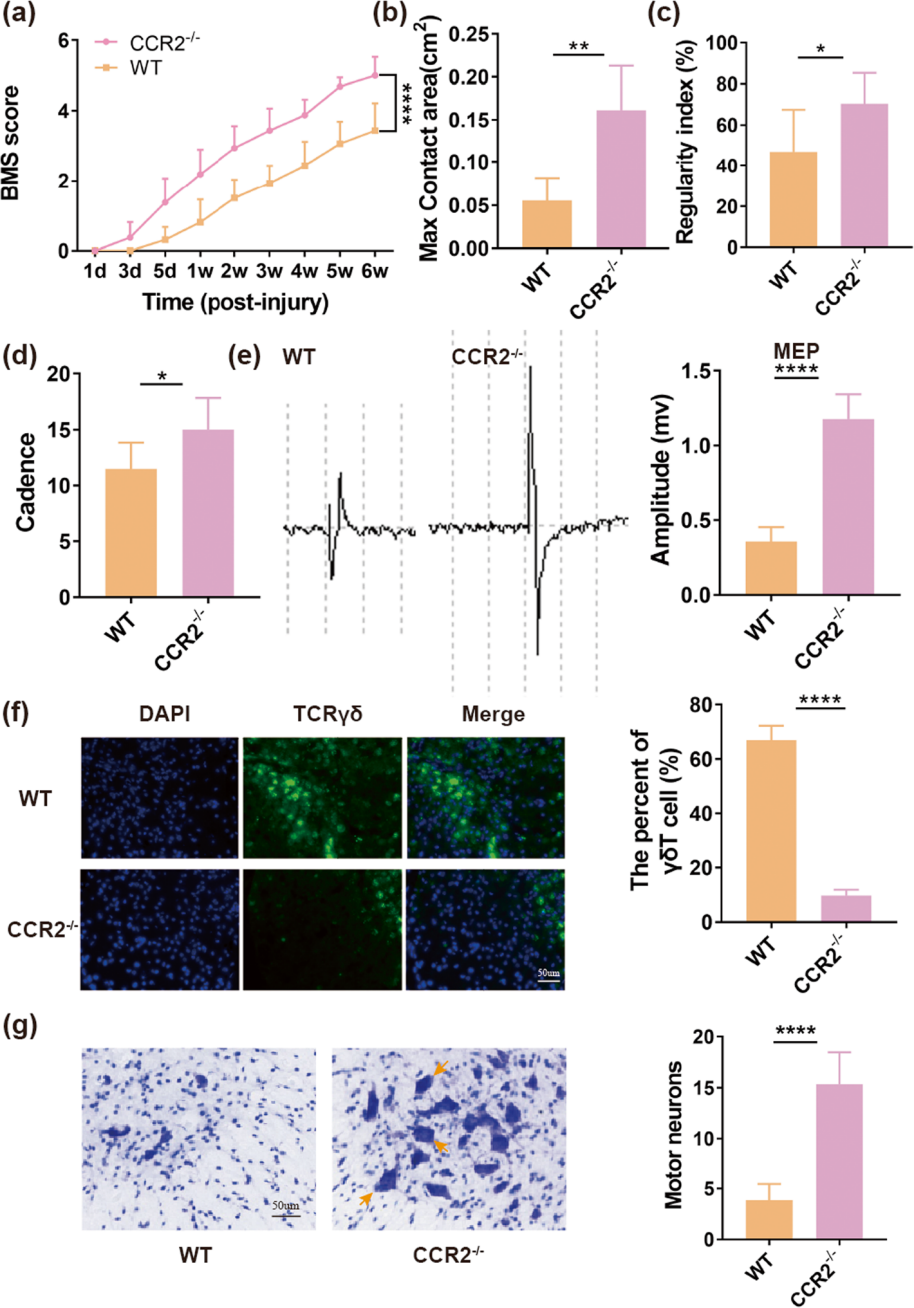
CCR2 plays a vital role during recruitment of γδ T cell to lesions. **a** BMS score of WT and CCR2^−/−^ mice at different time points after spinal cord injury (SCI) (*n* = 8 mice/per group). **b** Max contact area of WT and CCR2^−/−^ mice at 6 weeks after SCI (*n* = 8 mice/per group). **c** Regularity index of WT and CCR2^−/−^ mice at 6 weeks after SCI (*n* = 8 mice/per group). **d** Cadence of WT and CCR2^−/−^ mice at 6 weeks after SCI (*n* = 8 mice/per group). **e** Motor-evoked potential (MEP) recordings from WT and CCR2^−/−^ mice at 6 weeks post-surgery (*n* = 8 mice/per group). **f** Spinal sections from WT and CCR2^−/−^ mice after 1 day post-SCI were immunostained with anti-TCRγδ (green, a maker for γδ T cell) and the corresponding static histogram of percent of γδ T cell (γδ T cell/total cell) (*n* = 6 mice/per group). **g** Survival of motor neurons immunostained with Nissl staining in cross-section of injury cord epicenter at 6 weeks after SCI (*n* = 6 mice/per group). *P < 0.05; **P < 0.01; ****P < 0.0001

To investigate whether CCR2 mediated the recruitment of γδ T cells to the lesions after SCI, we drew a comparison on the aggregation number of γδ T cells at SCI site between CCR2^−/−^ and WT mice groups. Our results demonstrated that less numbers of γδ T cells were found at lesion epicenter site of CCR2^−/−^ mice group, compared with WT mice group (9.67 ± 2.16% vs. 67 ± 5.22%, *P* < 0.0001, Fig. 6f, *n* = 6 mice/per group). In addition, the BMS score, max contact area, regularity index, cadence, and MEPs also were assessed to compare the motor function recovery between CCR2^−/−^ and WT mice groups. We found that compared to WT mice, CCR2^−/−^ mice had earlier and faster BMS score recovery, and their mean BMS score was higher at 6 weeks after SCI (5 ± 0.54 vs. 3.44 ± 0.78, *P* < 0.0001, Fig. 6a, *n* = 8 mice/per group). Similar differences appeared in max contact area (0.16 ± 0.05 vs. 0.06 ± 0.03 cm^2^, *P* < 0.01, Fig. 6b), regularity index (70.41 ± 14.98% vs. 46.96 ± 20.47%, *P* < 0.05, Fig. 6c), cadence (15 ± 2.82 vs. 11.51 ± 2.34, *P* < 0.05, Fig. 6d), and MEPs (1.18 ± 0.17 vs. 0.36 ± 0.1 mv, *P* < 0.0001, Fig. 6e) between CCR2^−/−^ and WT mice groups at 6 weeks post-injury (*n* = 8 mice/per group). To further confirm this, the amount of survival motor neurons at lesion epicenter was measured by a Nissl staining at 6 weeks after SCI. Our results showed that CCR2^−/−^ mice had more motor neurons compared to WT mice (15.33 ± 3.14 vs. 3.83 ± 1.6, *P* < 0.0001, Fig. 6g, *n* = 6 mice/per group). According to those results, we knew that CCR2 mediated the recruitment of γδ T cells to lesions, and its depletion enhanced motor recovery.

### γδ T cells from CCR2^−/−^ or WT mice were transferred into TCRδ^−/−^ mice

**Fig. 7:**
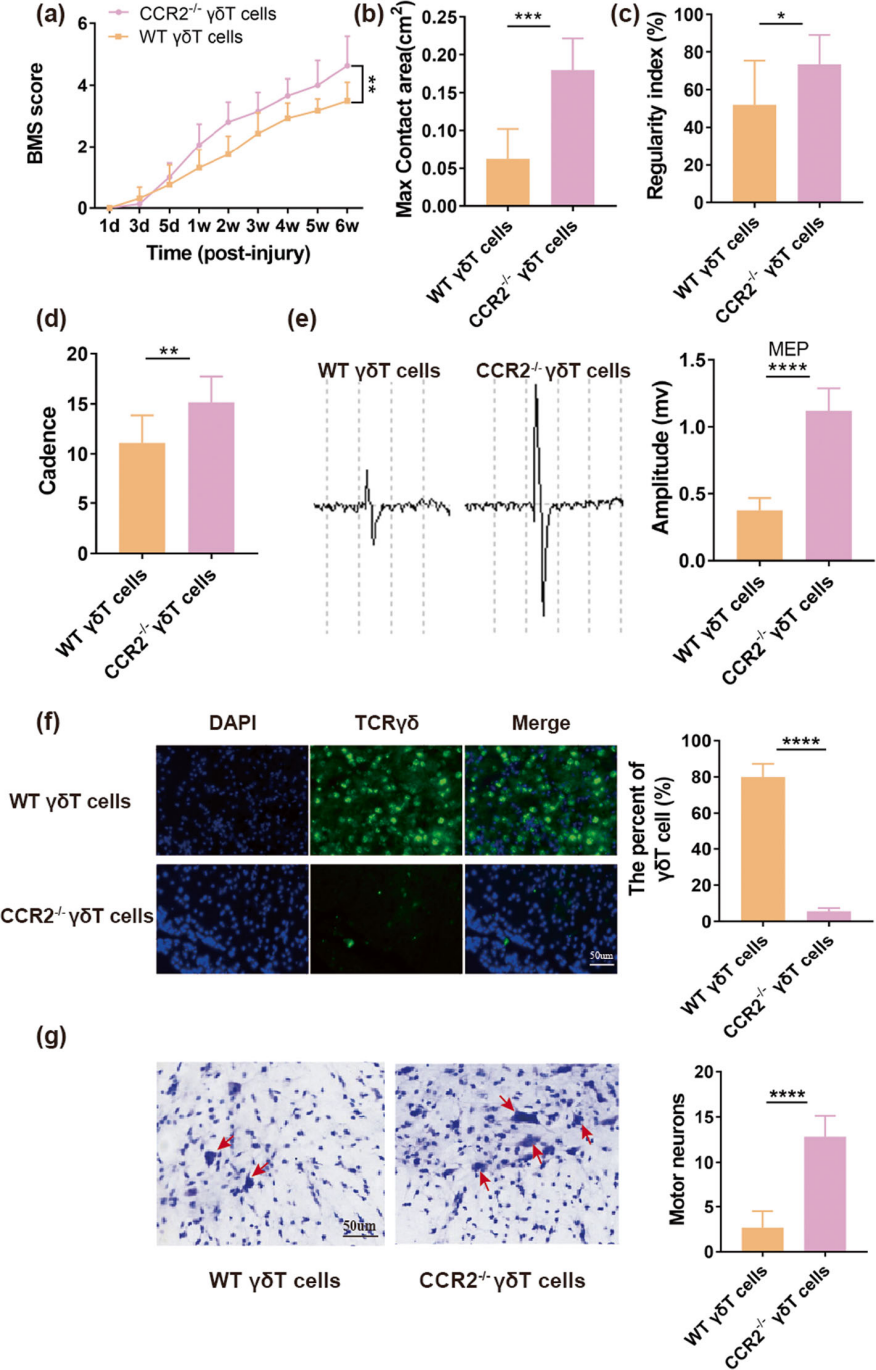
TCRδ^−/−^ mice were reconstituted with γδ T cells from CCR2^−/−^ and WT mice. **a** BMS score of WT and CCR2^−/−^ γδ T cells reconstituted mice at different time points after spinal cord injury (SCI) (*n* = 8 mice/per group). **b** Max contact area of WT and CCR2^−/−^ γδ T cells reconstituted mice at 6wk after SCI (*n* = 8 mice/per group). **c** Regularity index of WT and CCR2^−/−^ γδ T cells reconstituted mice at 6 weeks after SCI (*n* = 8 mice/per group). **d** Cadence of WT and CCR2^−/−^ γδ T cells reconstituted mice at 6wk after SCI (*n* = 8 mice/per group). **e** Motor-evoked potential (MEP) recordings from WT and CCR2^−/−^ γδ T cells reconstituted mice at 6 weeks post-surgery (*n* = 8 mice/per group). **f** Spinal sections from WT and CCR2^−/−^ γδ T cells reconstituted mice after 1 day post-SCI were immunostained with anti-TCRγδ (green, a maker for γδ T cell) and the corresponding static histogram of percent of γδ T cell (γδ T cell/total cell) (*n* = 6 mice/per group). **g** Survival of motor neurons immunostained with Nissl staining in cross-section of lesion epicenter at 6 weeks after SCI (*n* = 6 mice/per group). *P < 0.05; **P < 0.01; ***P < 0.001; ****P < 0.0001

To further confirm that CCR2 played a key part in the recruitment of γδ T cells to lesions after SCI, γδ T cells from CCR2^−/−^ or WT mice were transferred into TCRδ^−/−^ mice 1 day before SCI. Then BMS score, max contact area, regularity index, cadence, and MEPs were evaluated. Our results suggested that TCRδ^−/−^ mice transferred with γδ T cells from CCR2^−/−^ mice exhibited better motor function improvement than those reconstituted with γδ T cells from WT mice (*n* = 8 mice/per group), as shown in BMS score (4.63 ± 0.95 vs. 3.5 ± 0.6, *P* < 0.01, Fig. 7a), max contact area (0.18 ± 0.04 vs. 0.06 ± 0.04 cm^2^, *P* < 0.001, Fig. 7b), regularity index (73.45 ± 15.57% vs. 51.94 ± 23.51%, *P* < 0.05, Fig. 7c), cadence (15.18 ± 2.54 vs. 11.11 ± 2.77, *P* < 0.01, Fig. 7d), and MEPs (1.12 ± 0.17 vs. 0.38 ± 0.09 mv, *P* < 0.0001, Fig. 7e). Moreover, we performed anti-TCR-γ/δ immunostaining for the cross-section of injury site. We found that TCRδ^−/−^ mice reconstituted with γδ T cells from CCR2^−/−^ mice presented significantly less aggregation of γδ T cells at lesion epicenter site compared to those transferred with WT γδ T cells (80 ± 7.18% vs. 5.67 ± 1.75%, *P* < 0.0001, Fig. 7f, *n* = 6 mice/per group). To further identify this, a Nissl staining was performed to assess the number of survival motor neurons of lesion epicenter in cross section of spinal cord at 6 weeks post-injury. Our results demonstrated that more motor neurons were found in TCRδ^−/−^ mice reconstituted with γδ T cells from CCR2^−/−^ mice than in those transferred with WT γδ T cells (12.83 ± 2.32 vs. 2.67 ± 1.86, *P* < 0.0001, Fig. 7g, *n* = 6 mice/per group). Those results indicated again that CCR2 was involved in the recruitment of γδ T cells to spinal cord lesions.

**Fig. 8:**
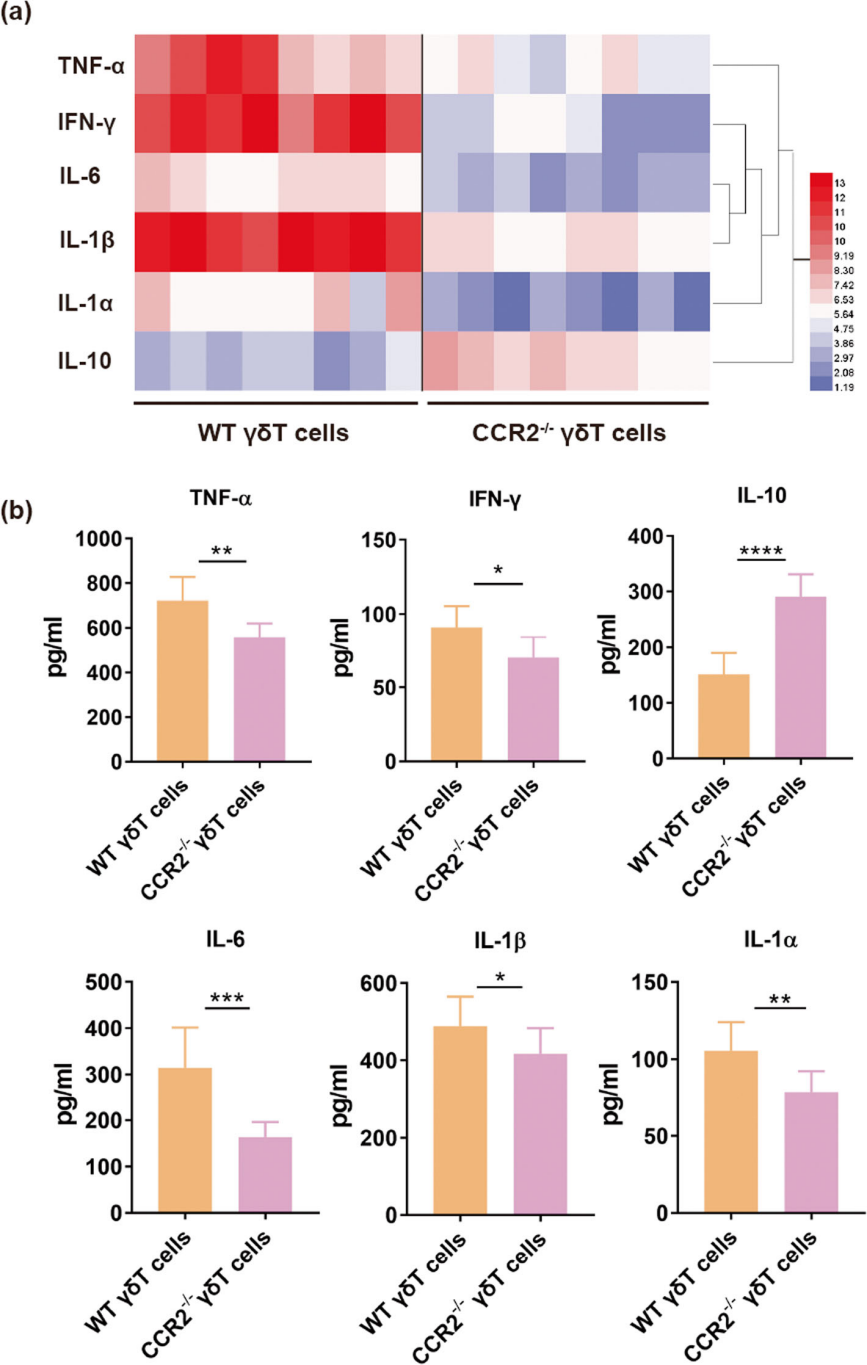
Comparison of level of cytokines between TCRδ^−/−^ mice reconstituted with CCR2^−/−^ γδ T cells and TCRδ^−/−^ mice transferred with WT γδ T cells at 1 day post-spinal cord injury (SCI). **a** Luminex analysis system (Bio-Rad, Hercules, CA) was used to analyze inflammatory protein level in spinal cord tissues at 1 day after SCI (*n* = 8 mice/per group). Different colors from low (blue) to high (red) represented the protein expression level (fold change). **b** Compared to WT γδ T cells reconstituted group, pro-inflammatory cytokines including TNF-α, IFN-γ, IL-1β, IL-1α, and IL-6, were significantly down-regulated and anti-inflammatory IL-10 was upregulated in CCR2^−/−^ γδ T cells transferred group (*n* = 8 mice/per group). *P < 0.05; **P < 0.01; ***P < 0.001; ****P < 0.0001

Additionally, to evaluate the expression of pro-inflammation cytokines, we conducted inflammatory protein analysis on spinal samples of TCRδ^−/−^ mice reconstituted with CCR2^−/−^ γδ T cells or WT γδ T cells at 1 day after SCI (Fig. 8a, *n* = 8 mice/per group). Meanwhile, the secreted level of inflammatory makers such as TNF-α, IFN-γ, IL-6, IL-1β, IL-α, and IL-10 were also measured at 1d after SCI. We found that pro-inflammatory-associated cytokines including TNF-α, IFN-γ, IL-17, IL-6, IL-1β, and IL-α were downregulated and anti-inflammatory IL-10 was upregulated in TCRδ^−/−^ mice reconstituted with CCR2^−/−^ γδ T cells after SCI compared with those mice transferred with WT γδ T cells (Fig. 8b, *n* = 8 mice/per group). Above results suggested that CCR2 depletion reduced the recruitment of γδ T cells to SCI site, and further decreased the production of pro-inflammatory cytokines.

## Discussion

Neurological damage due to SCI is a serious central nervous system disease that is exacerbated by the infiltration of immune cells and the inflammatory response around the injury site [23]. Therefore, investigating the pathological mechanism of SCI is urgently needed for the development of a more effective treatment. In the current study, our results showed that γδ T cells, especially CCR2^+^ γδ T cells, were recruited at the injury site after SCI, which hampered motor functional improvement. Amounts of CCL2 were secreted 12 h to 1 day after SCI. Inactivation of CCL2 or blocking of CCR2 lead to the decreased recruitment of γδ T cells at SCI site and neurological function improvement compared to WT mice, further confirming a vital role of CCL2-CCR2 signaling during recruitment of γδ T cells to SCI site. The results of γδ T cells from CCR2^−/−^ or WT mice transferred to TCRδ^−/−^ mice further supported our assumption. Collectively, our results demonstrated a pathological mechanism of SCI, defined the importance of CCL2-CCR2 signaling in the recruitment of γδ T cells to SCI site, and provided a novel potential therapeutic target.

γδ T cells are a subset of T lymphocytes that are unevenly distributed over the tissues of the body. Moreover, γδ T cells perform different functions when faced with different diseases. For instance, γδ T cells orchestrated the progress of immune responses through producing large amount of cytokines and those cells were highly lethal to infected cells during infectious diseases [24]. However, γδ T cells secreted a great deal of pro-inflammation cytokines that induced the deterioration of lesions in an experimental autoimmune encephalomyelitis (EAE) model [25]. In addition, γδ T cells also exerted different functions as shown in various tumor models [26, 27]. In the present study, our results showed that depletion of γδ T cells contributed to the improvement of neurological functions after SCI. Furthermore, we also found that γδ T cells, especially CCR2^+^ γδ T cells, were recruited at SCI site, and the reconstitution of TCRδ^−/−^ mice with γδ T cells from CCR2^−/−^ mice rather than WT mice improved the recovery of hind motor function. Those were consistent with the view that CCR2 may play an important role during the recruitment of γδ T cells to lesions after SCI.

CCL2 not only exhibits a good chemotaxis effect on immune cells but also regulates the activation and recruitment of macrophages and lymphocytes in many CNS diseases [28]. Previous evidence suggested that the expression level of CCL2 was elevated, and mainly secreted by activated microglia and astrocytes in neurological inflammatory responses [29, 30]. A similar result found in our study showed that the expression level of CCL2 increased significantly at 12 h to 1 day after SCI. Additionally, considerable evidence demonstrated that knock-out of CCL2 resulted in the decreased aggregation of M1 macrophages at the lesions in EAE mice models, and further slowed down the progression of neurological impairment and improved prognosis of the disease [31]. In this study, our results revealed that inactivation of CCL2 led to the reduced recruitment of γδ T cells to the site of SCI, and further enhanced neurological function recovery. This result indicated that CCL2 was an indispensable factor for the recruitment of immune cells such as γδ T cells to injury site.

CCL2 preferentially binds to the CCR2 found in a variety of tissues such as thymus, lungs, and liver [10]. Mounting data demonstrated that binding of CCL2 to the associated CCR2 contributed to the recruitment and invasion of immune cells such as macrophages and T lymphocyte to the lesions [32–34]. In this study, our results suggested that high levels of CCL2 and CCR2 were found in SCI, per mouse models. Meanwhile, to confirm the importance of CCL2/CCR2 axis to inflammation-induced recruitment of γδ T cells to injury site, we performed a comprehensive experimental comparison between CCL2^−/−^ or CCR2^−/−^ mice and WT mice. Our results showed that compared to WT mice, γδ T cell recruitment to the SCI site was significantly reduced, and improvement of neurological function was obviously enhanced in CCL2^−/−^ and CCR2^−/−^ mouse models. This revealed that CCL2/CCR2 signaling plays a key role in the recruitment of γδ T cells to SCI site, and blocking of CCL2/CCR2 axis contributes to recovery of hind motor function. Therefore, the CCL2/CCR2 signaling may be considered a potential therapeutic target to prevent progression of SCI.

Previous several studies indicated that CCL2/CCR2 may be mainly associated with the neuroinflammatory response to the primary injury, and early production of chemokines after SCI that precedes the invasion of neutrophils, monocytes, and T cells into the injury spinal cord thereby mediating inflammatory development within the lesion cord site as a recruitment mechanism of circulating immune cells [35–37]. Furthermore, neuroinflammation leads to secondary injury and decreased recovery of neurological function after SCI. Taken together, CCL2/CCR2 signaling probably play an important role in recovery procession of neurological function after SCI. In this study, our results indicated that CCL2/CCR2 signaling mediated the recruitment of γδ T cells to injury site, and γδ T cell recruitment to SCI site promotes inflammatory response and exacerbates neurological impairment.

γδ T cells can be classified as either IFN-γ or IL-17 producers according to their biological characteristics [38]. IL-17 is a key cytokine that regulates inflammatory progress, and serves as a communication bridge between immune cells and tissues [39]. Previous evidence demonstrated that the level of IL-17 was elevated after SCI, thus promoting an inflammatory response, enlarging the area of lesions, and further weakening neurological recovery [40]. However, IL-17 does not directly activate astrocytes that secrete amounts of pro-inflammation cytokines, but does through synergistic effects with other cytokines such as IL-6 and TNF-α [41]. Additionally, our results showed that large numbers of γδ T cells were recruited at SCI site, which aggravated the inflammatory response and hampered motor function improvement. Taken together, we made an initial conclusion that γδ T cell recruitment to SCI site may produce amounts of IL-17 that promote progression of neuroinflammation and block improvement of neurological functions. However, further studies should be performed to confirm this conclusion.

## Conclusions

In general, as shown in our results, γδ T cell recruitment to SCI site promotes inflammatory response and exacerbates neurological impairment. Furthermore, CCL2/CCR2 signaling is a key recruitment mechanism of γδ T cells to injury site and may be a potential treatment target for future SCI.

## Supplementary Information

**Additional file 1.**

## Data Availability

All the datasets and materials supporting the conclusions of this article are presented in the manuscript.

## Abbreviations

SCI: Spinal cord injury
CCL2: C-C motif chemokine ligand 2
CCR2: C-C chemokine receptor type 2
ELISA: Enzyme-linked immunosorbent assays
PFA: Paraformaldehyde
CSF: Cerebrospinal fluid
QRT-PCR: Quantitative real-time polymerase chain reaction
MEPs: Motor-evoked potential testing
MACS: Magnetic-activated cell sorting
SD: Standard difference
SEM: Standard error of mean
BMS: Basso Mouse Scale
EAE: Experimental autoimmune encephalomyelitis
