# Supplementary material for: Recruitment of γδ T cells to the lesion via the CCL2/CCR2 signaling after spinal cord injury
Source: J Neuroinflammation. 2021 Mar 2;18:64. doi: 10.1186/s12974-021-02115-0 (PMC7927238; doi:10.1186/s12974-021-02115-0)
Supplement: Supplementary file 1 — Additional file 1. [file 12974_2021_2115_MOESM1_ESM.doc]

CCL2 Sense: AGTTGACCCGTAAATCTGAAGC

Anti-Sense: AGGCATCACAGTCCGAGTC

CCR2 Sense: GAGTGAGAAGGAGGAGATATGC

Anti-Sense: AACACAGATAGGAGAAGGAACC

GAPDH Sense:GCCTTCCGTGTTCCTACC

Anti-Sense:GCCTGCTTCACCACCTTC
